# Low-Level Laser Application in the Early Myocardial Infarction Stage Has No Beneficial Role in Heart Failure

**DOI:** 10.3389/fphys.2017.00023

**Published:** 2017-01-30

**Authors:** Martha T. Manchini, Ednei L. Antônio, José Antônio Silva Junior, Paulo de Tarso C. de Carvalho, Regiane Albertini, Fernando C. Pereira, Regiane Feliciano, Jairo Montemor, Stella S. Vieira, Vanessa Grandinetti, Amanda Yoshizaki, Marcio Chaves, Móises P. da Silva, Rafael do Nascimento de Lima, Danilo S. Bocalini, Bruno L. de Melo, Paulo J. F. Tucci, Andrey J. Serra

**Affiliations:** ^1^Laboratory of Biophotonic, Nove de Julho University, São PauloSão Paulo, Brazil; ^2^Laboratory of Cardiac Physiology, Federal University of São PauloSão Paulo, Brazil; ^3^Medicine Program, Nove de Julho UniversitySão Paulo, Brazil; ^4^Translational Physiology Laboratory, Brazil Physical Education and Aging Science Program, São Judas Tadeu UniversitySão Paulo, Brazil

**Keywords:** angiogenesis, cardiac remodeling, cardiac performance, low-level laser therapy, myocardial infarction

## Abstract

Low-level laser therapy (LLLT) has been targeted as a promising approach that can mitigate post-infarction cardiac remodeling. There is some interesting evidence showing that the beneficial role of the LLLT could persist long-term even after the end of the application, but it remains to be systematically evaluated. Therefore, the present study aimed to test the hypothesis that LLLT beneficial effects in the early post-infarction cardiac remodeling could remain in overt heart failure even with the disruption of irradiations. Female Wistar rats were subjected to the coronary occlusion to induce myocardial infarction or Sham operation. A single LLLT application was carried out after 60 s and 3 days post-coronary occlusion, respectively. Echocardiography was performed 3 days and at the end of the experiment (5 weeks) to evaluate cardiac function. After the last echocardiographic examination, LV hemodynamic evaluation was performed at baseline and on sudden afterload increases. Compared with the Sham group, infarcted rats showed increased systolic and diastolic internal diameter as well as a depressed shortening fraction of LV. The only benefit of the LLLT was a higher shortening fraction after 3 days of infarction. However, treated-LLLT rats show a lower shortening fraction in the 5th week of study when compared with Sham and non-irradiated rats. A worsening of cardiac function was confirmed in the hemodynamic analysis as evidenced by the higher LV end-diastolic pressure and lower +dP/dt and −dP/dt with five weeks of study. Cardiac functional reserve was also impaired by infarction as evidenced by an attenuated response of stroke work index and cardiac output to a sudden afterload stress, without LLLT repercussions. No significant differences were found in the myocardial expression of Akt_1_/VEGF pathway. Collectively, these findings illustrate that LLLT improves LV systolic function in the early post-infarction cardiac remodeling. However, this beneficial effect may be dependent on the maintenance of phototherapy. Long-term studies with LLLT application are needed to establish whether these effects ultimately translate into improved cardiac remodeling.

## Introduction

Myocardial infarction (MI) is a major cause for heart failure (HF) development (Yancy et al., 2013). Data are showing that three million people are affected by MI in the USA, and more than 400.000 new cases are reported for each year. In fact, ~50% of patients will die within 5 years, and 40% die 12 months after the first HF hospitalization (Kolseth et al., 2014).

The acute MI triggers an adverse process known as cardiac remodeling, in which there is left ventricular (LV) dilation and enlargement of the ischemic tissue (Serra and Tucci, 2016). Moreover, an impaired LV systolic and diastolic function and a reduced myocardial inotropism are well-documented findings (dos Santos et al., 2013; Antonio et al., 2015). Several mechanisms are shown to be implicated in cardiac remodeling, including adrenergic hyperactivity, renin-angiotensin-aldosterone system, apoptosis, autophagy, fibrosis, inflammation, oxidative stress, calcium handling abnormalities, and metabolic dysfunction (Whelan et al., 2010; Carlos et al., 2016; Ziff et al., 2016). Moreover, post-infarction cardiac remodeling is associated with a higher prevalence of cardiac rupture, arrhythmias, and formation of aneurysms. In the long term, there is the development of HF and sudden death (Whelan et al., 2010; Ziff et al., 2016).

Several interventions have been proposed to alleviate cardiac remodeling to prolong or prevent the development of HF (Carlos et al., 2016). However, current therapies have shown only modest results in survival or potential adverse properties (Yancy et al., 2013; Grosman-Rimon et al., 2016). In latest years, experimental studies have punctuated that the low-level laser therapy (LLLT) may be a promising approach to modulate various biological processes (Albertini et al., 2008; Pires et al., 2011). The LLLT stimulates photoreceptors in the mitochondrial respiratory chain, resulting in increased ATP, increased growth factor secretion and tissue healing (Tuby et al., 2006; Huang et al., 2011; Peplow et al., 2012). A cardiac LLLT effect has been reported for over 10 years, in which infarcted rats showed a lower myocardial necrosis (Oron et al., 2001b), LV dilatation (Ad and Oron, 2001; Yaakobi et al., 2001), and most favorable milieu to prevent scar disruptions (Whittaker and Patterson, 2000) with LLLT. More recently, our group demonstrated reduced infarct size, attenuated the systolic dysfunction and beneficial modulates inflammation and expression of vasoactive peptides in rats submitted to LLLT (Manchini et al., 2014).

In a recent systematic review, we have reported that many studies have only assessed the LLLT role at MI early stage, in which data reporting effects on the progression to HF are limited (Carlos et al., 2016). Moreover, an intriguing is issue shown to be an attenuated cardiac remodeling in animals submitted to LLLT only at the initial phase of injury. In this regard, it has been shown benefits of LLLT after several weeks post-MI, e.g., decreased infarct size and cardiac dilation (Oron et al., 2001b; Yaakobi et al., 2001). Although these data indicate that the benefits of LLLT in the acute phase of MI may persist in overt HF, there are some limitations that should be considered: (i) there is no blinding for the experimental group or outcomes. A more suitable method would be to blind the infarct size and LLLT; (ii) inclusion/exclusion criteria has not been stated (e.g., animals with similar infarct sizes). The control of infarct size it seems to be a key issue because the remodeling is intensified on larger infarctions. Thus, it is doubtful to consider a beneficial cardiac remodeling LLLT effect because of the intragroup infarct size variability; (iii) there is only cross-sectional design studies, and the causality results cannot be determined. Therefore, this study was designed to determine whether LLLT application benefits at the MI early stage remains in overt HF same with disruption of treatment.

## Materials and methods

### Animals and experimental design

This study was carried out in accordance with the recommendations of Guide for the Care and Use of Laboratory Animals published by the US National Institutes of Health (NHI, no. 85-23, revised 1996). The protocol was approved by the Institutional Research Ethics Committee of the Nove de Julho University, São Paulo, Brazil (number: 0015–2012). Experiments were performed under ketamine (50 mg/kg), and Xylazine (10 mg/kg) mixture anesthesia, and efforts were made to minimize the suffering of animals.

**Figure 1:**
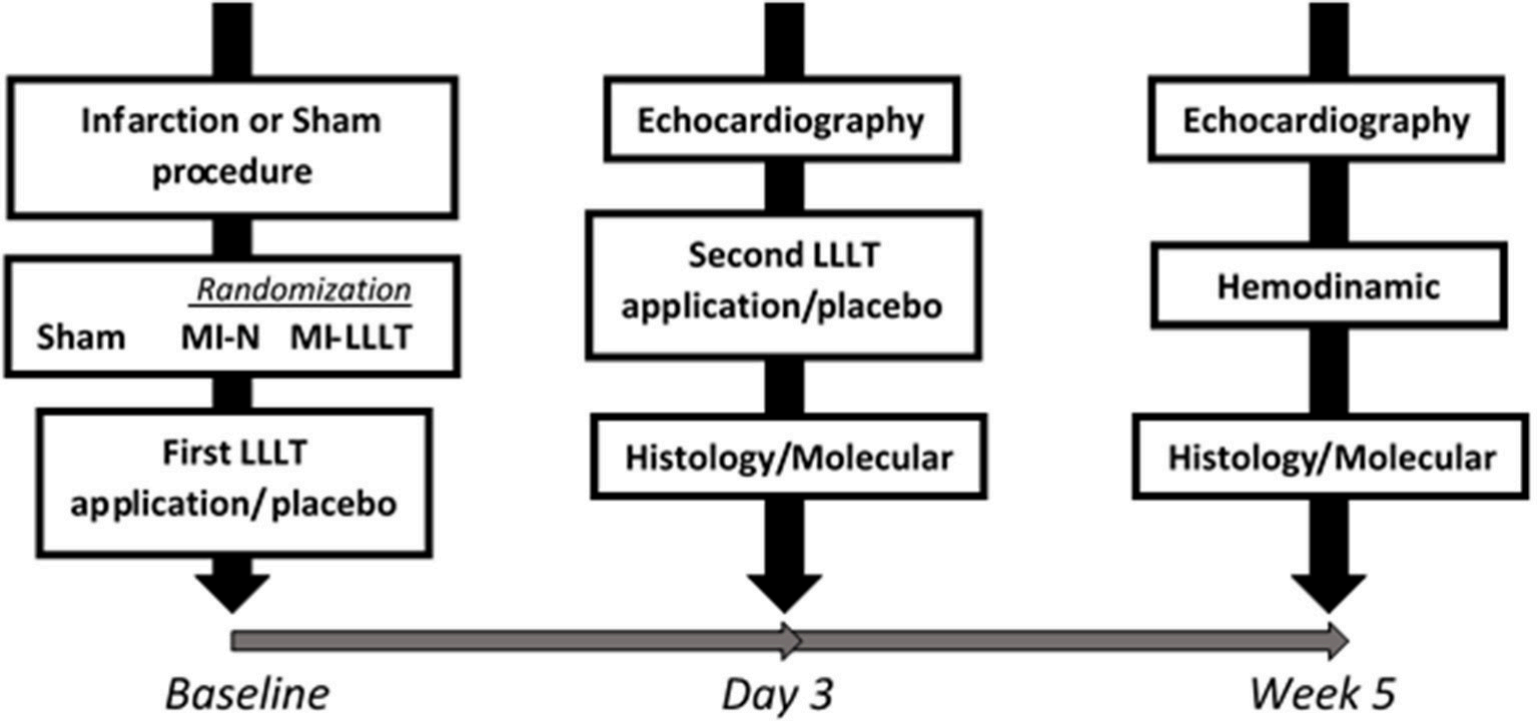
**Experimental design protocol**.

Figure 1 illustrates the experimental design. Forty-seven female Wistar rats weighing 250–280 g were assigned to LLLT or non-LLLT. The MI was produced by permanent arterial coronary occlusion, and then rats were randomized to one of the following groups: infarcted rats non-treated with LLLT (MI-N, *n* = 7); infarcted rats submitted to LLLT (MI-LLLT, *n* = 6). Sham rats (*n* = 8) were operated upon similarly, although the coronary occlusion was avoided. The Sham and MI-N groups were submitted to a similar LLLT procedure, yet the device was kept off (placebo). Echocardiographic analyses were carried out on 3 days and 5 weeks post-infarction. We have included in the study only rats with large infarcts, which showed be defined on the 3rd-day post-infarction as a size ≥ 37% of LV (dos Santos et al., 2013). At the end of the 5-week, rats were euthanized by decapitation according to a protocol detailed elsewhere (AVMA Panel on Euthanasia. American Veterinary Medical Association, 2001). The infarct scar was removed from LV, and remote myocardial tissue was immediately stored in a cryogenic tube and kept frozen in liquid nitrogen for molecular analysis.

To date, nine rats died in coronary occlusion surgery, four during the peri-operative period, and two in the hemodynamic evaluation. We excluded 11 rats because they had infarct sizes < 37%.

### MI model

The MI was induced according to a well-established technique (Antonio et al., 2015). Briefly, under anesthesia and artificial ventilation (Harvard Rodent Ventilator, Model 863; Harvard Apparatus, Holliston, MA, USA), a left thoracotomy was performed. The heart was exteriorized, and the left anterior descending coronary artery was occluded near its origin with 6-0 polypropylene. The heart was rapidly returned to its original position and the thorax closed.

### Phototherapy

Aluminum Indium Gallium Phosphorus—AlGaInP (Twin Laser—MM Optics, São Carlos, SP, Brazil) was used for irradiation under the parameters in Table 1. After thoracotomy, the coronary occlusion was carried out as describe above, and the heart was put in the chest to recover itself for 60 s and then the organs was externalized. The laser/placebo was applied directly to the myocardial tissue targeting infarcted area. In the 3rd day, rats were anesthetized, and a new thoracotomy was performed at the same surgical site to heart exteriorization and laser/placebo application. Sham group was exposed to all experimental procedures, though the LLLT device was off.

**Table 1:** **Protocol of LLLT irradiation**.

**Active laser medium**	**AlGaInP**
Power (mW)	15
Wave-length (nm)	660
Power density (W/cm^2^)	0.37
Density energy (J/cm^2^)	27.3
Spot size (cm^2^)	0.04
Time per point (sec)	73
Total energy per treatment (J)	1.1
Number of treatments (once a day)	2
Application mode	Punctual in heart

### Echocardiography

Rats were anesthetized as described above (K-X mixture) and LV echocardiography was performed using a 12-MHz transducer connected to an HP Sonos-5500 (Hewlett–Packard, Palo Alto, CA, USA). The infarct size was evaluated on transverse 2-dimensional view and reported as percent of the LV perimeter on the basal, mid transversal, and apical planes (Sofia et al., 2014). The MI was defined as the presence of a segment with increased echogenicity and modification in myocardial thickening or systolic movement (hypokinesia, akinesia, or dyskinesia). Systolic function was analyzed by the fractional shortening (Serra et al., 2010). Diastolic function was not evaluated owing to the fusion of the A and E waves.

### LV hemodynamic study and afterload stress

Immediately after echocardiography, baseline hemodynamic evaluation was performed under adjusted anesthesia (K-X mixture) and oxygen-enriched ventilation with a closed chest. The left femoral vein was accessed for drug administration, and a 2-F gauge Millar catheter-tip micromanometer (model SPR-320, Millar Instruments, Houston, TX, USA) was inserted into the right carotid artery into the LV cavity. Moreover, an ultrasound flow probe (Transonic System Inc., Ithaca, NY, USA) was positioned in the ascending aorta. The following data were analyzed (Acknowledge software, Biopac System, Santa Barbara, CA, USA): LV systolic (SP) and end-diastolic pressures (EDP), rate of change of LV pressure (+dP/dt and −dP/dt), heart rate, and cardiac output (CO), and stroke volume (SV). Stroke work index (SWI) was stated as previously described (dos Santos et al., 2010). Thereafter, sudden LV afterload increases were achieved using a single phenylephrine in bolus injection (15–25 μg/kg, i.v.) (dos Santos et al., 2010).

### Biometric data

After hemodynamic analysis, hearts were quickly removed and weighed. Myocardial mass was indexed by body weight and used as a hypertrophy marker.

### Myocardial fibrosis

Hearts were removed in 3 days and 5 weeks after infarction or sham surgery and fixed in 4% buffered formaldehyde overnight. The LV fragments were washed with PBS, dehydrated through a graded series of ethanol, diaphonized with Xylol and embedded with paraplast. Samples were cut into 3 mm thick sections and stained with Masson's trichome. The fibrous tissue was evaluated in 6 randomized 40 x magnification using a Nikon Eclipse E200 microscope and Nikon Infinity Optical System (Kurobane Nikon Co., Tochigi, Japan), and Image Pro-Plus software, version 4.0 (Media Cybernetics Inc., Rockville, MD, USA).

### Western blot

Proteins were extracted from the LV remote area as previously described by us (Silva et al., 2014). Homogenate protein samples of 30 μg were subjected to SDS-PAGE in 10% polyacrylamide gel. Separated proteins were transferred onto hydrophobic polyvinylidene difluoride membranes (Hybond-P, Amersham Biosciences; Piscataway, J, USA), and the transfer efficiency was examined with 0.5% Ponceau S. The membranes were soaked in a blocking buffer (5% nonfat dry milk and 0.1% Tween 20 in PBS, pH 7.5) for 1 h at room temperature and then incubated overnight at 4°C with primary antibodies: rabbit anti-Akt1 (1:5000 dilution; Abcam, Cambridge, MA, USA); rabbit anti-phosphoSer473Akt1 (1:5000 dilution; Abcam, Cambridge, USA); goat anti-VEGF (1:1000; Abcam, Cambridge, MA, USA); anti-GAPDH (1:500; Santa Cruz Biotechnology, Santa Cruz, CA, USA). After overnight incubation, membranes were washed five times and then incubated for 1 h with horseradish peroxidase-conjugated goat anti-rabbit and rabbit anti-goat secondary antibodies (1:2000; Invitrogen, San Diego, CA, USA). Membranes were finally washed five times with blocking buffer and then rinsed twice in PBS. Bound antibody was detected by using chemiluminescence reagent for 1 min. The bands were imaged by using Amersham Imager 600 system (GE Health Care, Little Chalfont, UK). UK).

### Statistical analysis

Data were analyzed using GraphPad Prism software 5.0 (La Jolla, CA, USA). Shapiro-Wilk test was used to verify normality data. Levene test was applied to assess the equality of variances. One-way ANOVA complemented by Newman–Keuls *post hoc* was applied to detect differences between groups in cross-section analysis. Two-way repeated ANOVA complemented by Bonferroni *post hoc* was applied to paired data. Kruskal-Wallis followed by Dunn's multiple comparison tests were applied to non-normality data. Statistical significance was set at *p* ≤ 0.05. Data are expressed as mean ± SD.

## Results

### LLLT does not affect structural and functional abnormalities of LV

**Figure 2:**
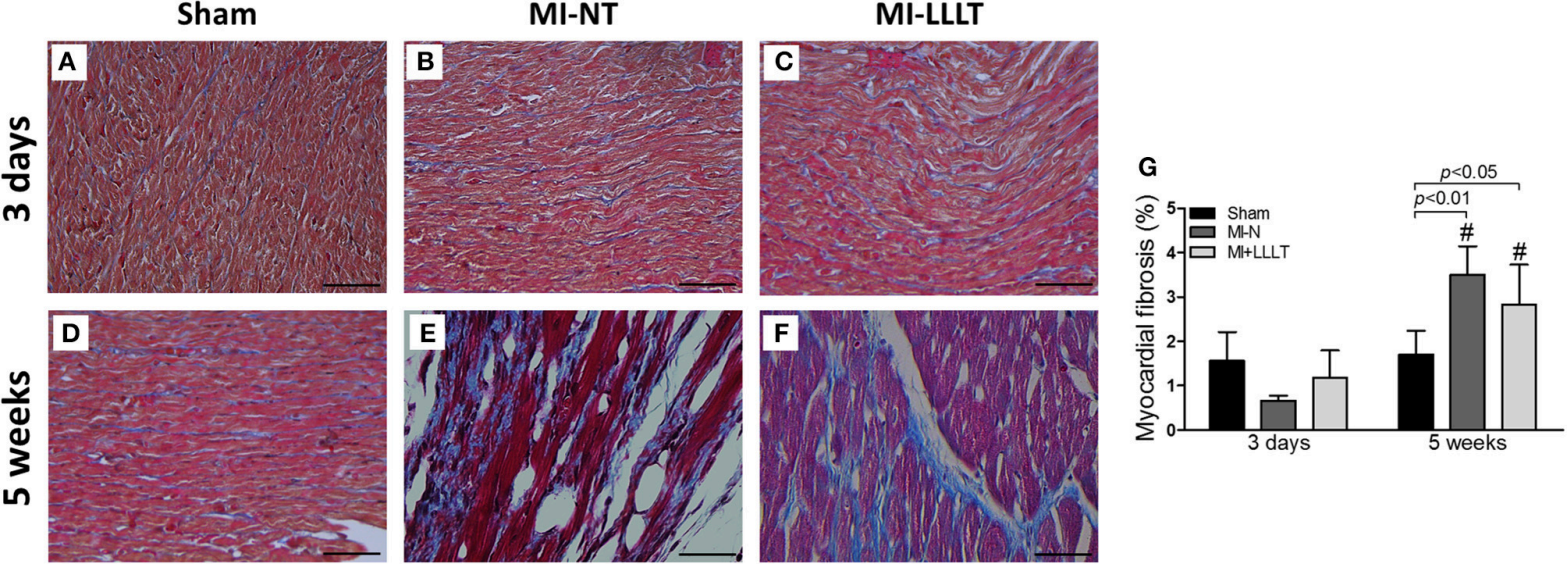
**Representative microphotographs of remote myocardium with 3 days** (**A**: Sham; **B**: MI-N; **C**: MI+LLLT) and 5 weeks (**D**: Sham; **E**: MI-N; **F**: MI+LLLT) after MI. Myocardial sections were stained with Masson's trichome. **(G)** Is representative of the statistical comparisons between experimental groups. Data are means ± SD (*n* = 4 per group). *P*-values were determined by one-way ANOVA and *post hoc* Newman-Keuls test. Magnification 40x (scale bar: 50 μm). ^#^*p* < 0.05 vs. 3 days.

The biometric data are shown in Table 2. Average body weight was similar between the three experimental groups on the 3rd day and 5 weeks of study. Infarcted rats showed a similar heart mass as well as heart mass-to-body weight ratio when compared with Sham rats, and phototherapy had no repercussion on heart mass. Besides, phototherapy also had no effect on infarct size. As evidenced in Figure 2, quantitative analysis for Masson trichome staining indicated no significant differences in the collagen content between experimental groups with 3 days post-MI. However, infarcted rats showed a significant increase of fibrosis over 5 weeks post-MI, in which LLLT had no significant effect.

**Table 2:** **Biometric data**.

	**3 days**			**5 weeks**		
	**Sham**	**MI-N**	**MI+LLLT**	**Sham**	**MI-N**	**MI+LLLT**
BW (g)	210 ± 29	192 ± 23	206 ± 30	239 ± 20	237 ± 17	245 ± 27
Heart mass (mg)	–	–	–	1004 ± 138	894 ± 186	1062 ± 447
Heart mass/BW ratio	–	–	–	4.3 ± 0.9	3.7 ± 0.7	4.2 ± 1.3
Infarct size (%)	–	46 ± 16	44 ± 7	–	50 ± 7	49 ± 5

*BW, body weight. Two-way repeated ANOVA was applied for body weight and infarct size analysis. ANOVA one way was applied for heart mass and heart mass index. There were no significant differences as a result of time or phototherapy. Data are expressed as mean ± SD*.

**Figure 3:**
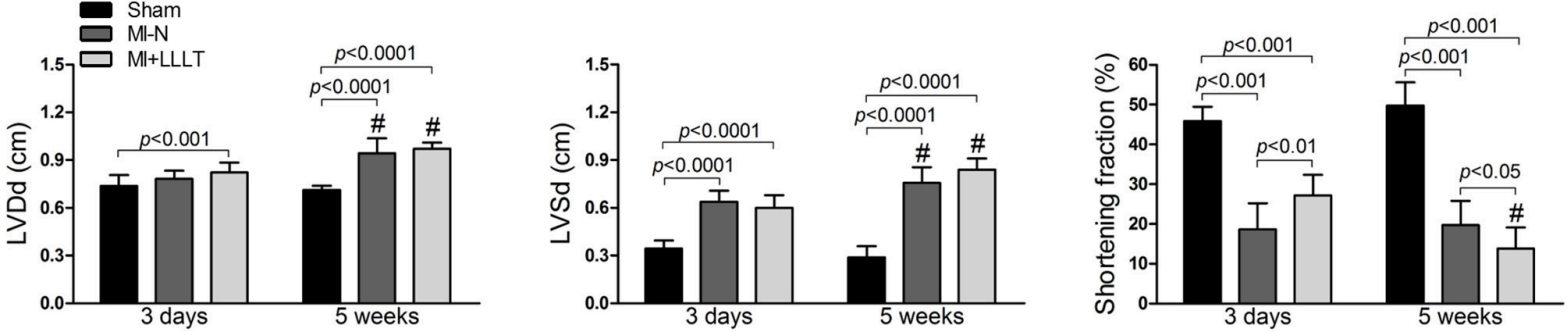
**Effects of LLLT in the ventricular cavity and fractional shortening 3 days and 5 weeks after MI (Sham, *n* = 8; MI-N, *n* = 7; MI+LLLT, *n* = 6)**. Compared with the Sham group, the left ventricular end diastolic diameter (LVDd) were significantly augmented in 3 days (MI+LLLT group) and 5 weeks (MI-N and MI+LLLT). The left ventricular end systolic diameter (LVSd) was higher in all infarcted groups compared with Sham group (3 days and 5 weeks). The fractional shortening of the MI-LLLT group was significantly higher than MI-N group on 3 day, however MI+LLLT showed significantly lower LV performance in 5 weeks when compared with Sham and MI-N groups. Data are means ± SD. *P*-values were determined by two-way repeated ANOVA complemented by Bonferroni *post-hoc*. ^#^*p* < 0.05 vs. 3 days.

As seen in Figure 3, there was LV dilatation with only 3 days post-infarction, in which diastolic diameter was significantly higher in MI+LLLT group while the systolic diameter was higher in all infarcted groups when compared with Sham group. At the end of the 5-week experimental period, both diastolic and systolic diameters were shown to be significantly increased in all infarcted groups when compared with Sham group. The LV systolic dysfunction was apparent early as the 3rd-day post-infarction, as evidenced by a minor fractional shortening. The beneficial role of LLLT was only noticed in the early (3 days) post-infarction cardiac remodeling, in which the fractional shortening of the MI-LLLT group was significantly higher than MI-N group. On the other hand, treated-LLLT rats show a lower LV performance in the 5th week of study when compared with Sham and MI-N rats.

**Figure 4:**
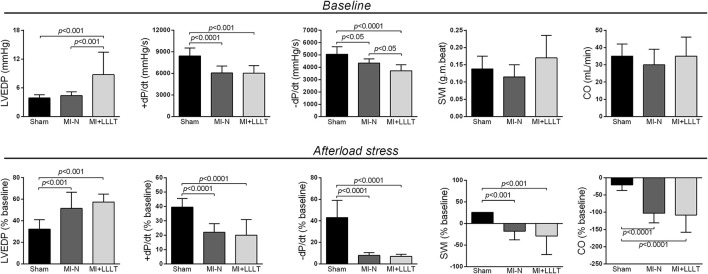
**Repercussion on baseline hemodynamic and sudden afterload after 5 weeks of MI (Sham, *n* = 8; MI-N, *n* = 7; MI+LLLT, *n* = 6)**. Data are means ± SD. *P*-values were determined by one-way ANOVA and *post hoc* Newman-Keuls test. LVEDP: left ventricular end-diastolic pressure; +dP/dt: maximum positive time derivative of developed pressure; −dP/dt: maximum negative derivative of developed pressure; SWI: stroke work index CO: cardiac output.

Afterward second echocardiographic analysis, an invasive hemodynamic evaluation was carried out to determine LV ejection performance. As reported in Figure 4, data also indicate deteriorating LV function, in which +dP/dt and −dP/dt values were significantly lower in MI-N and MI-LLLT groups compared with Sham group under basal conditions. In addition, a higher EDP was reported only for MI-LLLT group, which also showed a more marked reduction on −dP/dt when compared to MI-N and Sham group. The LV ejection parameters from all infarcted groups did not differ significantly from those of the Sham group when evaluated under basal conditions, as evidenced by SWI and CO. These findings led to analyze the cardiac functional reserve during sudden afterload stress as a result of *in bolus* phenylephrine injection. For suitable homogenization, we carried out experiments to raise the blood pressure of 50–70% over the baseline level (dos Santos et al., 2010). This afterload range was accompanied by a higher increase of +dP/dt and −dP/dt in Sham group than in all infarcted groups. Furthermore, CO decreased more dramatically in all infarcted groups when compared with Sham group. Ultimately, Sham rats showed SW increase, whereas the SW was remarkably reduced in all infarcted rats.

### Survival/angiogenesis factors are not affected by MI or LLLT

**Figure 5:**
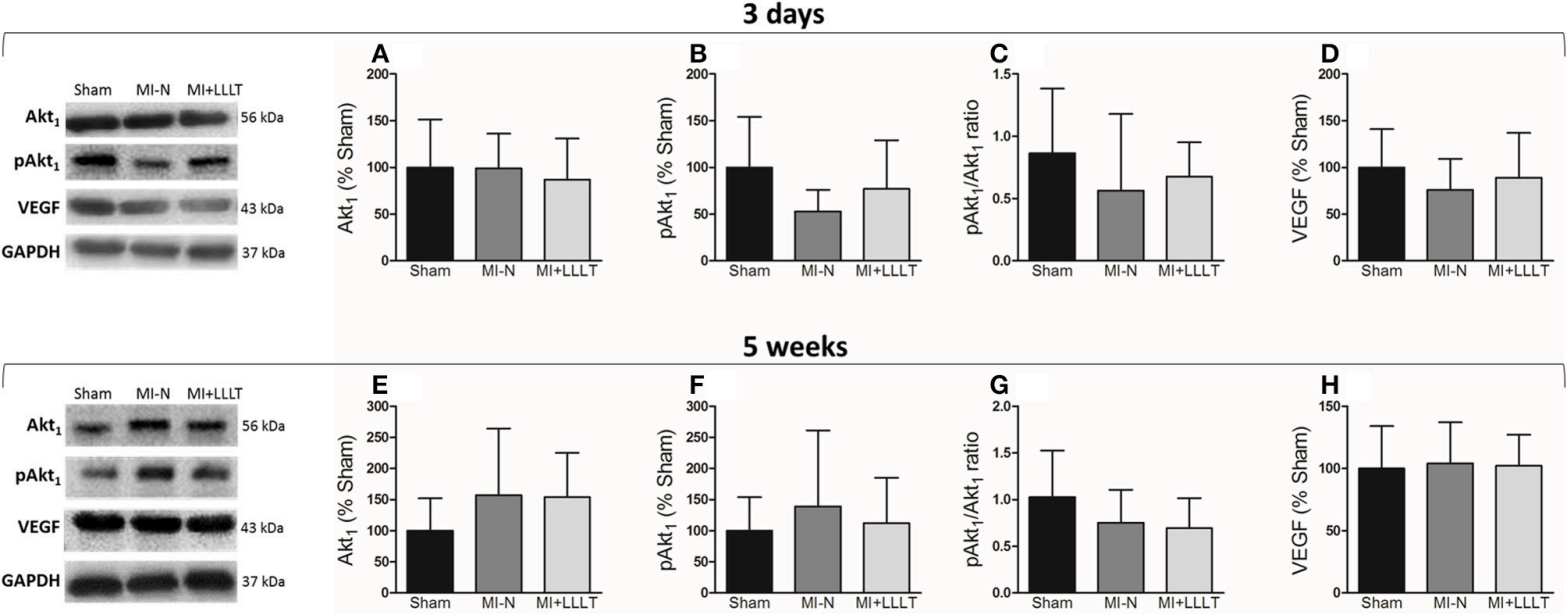
**The protein expression by western blot in the remote myocardium with 3 days and 5 weeks after MI. (A,E)** Protein expression of Akt_1_. **(B,F)** Protein expression of pAkt_1_. **(C,G)** Ratio of pAkt_1_/ Akt_1_. **(D,H)** Protein expression of VEGF. All values were normalized to levels of GAPDH (Sham, *n* = 8; MI-N, *n* = 7; MI+LLLT, *n* = 6). Data are means ± SD. *P*-values were determined by one-way ANOVA and *post hoc* Newman-Keuls test.

It has been postulated that the cardioprotective effects of LLLT shown to be associated with increased angiogenesis, and this action is linked to modulation of vascular endothelial growth factor (VEGF) (Tuby et al., 2006, 2008). Thus, we have investigated the Akt_1_/VEGF pathway in the remote myocardial after 5 weeks following injury. Data in Figure 5 indicate that the MI and phototherapy did not affect the expression of the total Akt_1_, Akt_1_ phosphorylated at Serine 473 and Akt_1_/pAkt_1_ ratio, which is a marker of its activity. Notwithstanding, VEGF expression was also not significantly different between the experimental groups.

## Discussion

Data showing that LLLT application only at the MI early stage could result in a long-term beneficial effect on cardiac remodeling are intriguing. In fact, LLLT action has been achieved until several weeks after discontinuing of the irradiation, e.g., a minor infarct size (Oron et al., 2001b).

We showed here that the LLLT improved LV systolic function only 3 days post-infarction, which confirms previous data from our lab (Manchini et al., 2014). On the other hand, we have not reported a cardioprotective LLLT role during evolution to overt HF, as illustrated by the no effect on infarct size, cavity dilation and LV systolic performance at the end of the study. Yang et al. (2011) have published similar findings in rats subjected to LLLT with up to 72 h post-infarction. Accordingly our data, these authors also reported no beneficial LLLT effect in LV diastolic and systolic diameter as well as LV performance on echocardiographic analysis. We advance these findings to explore whether the LLLT could increase functional heart reserve for an increased LV afterload. In fact, a minor functional LV reserve shown be a marker for cardiac remodeling progression (Fletcher et al., 1981), in which it can be the result of a decreased myocardial inotropism at a given loading level (Francis et al., 2001). As illustrated in Figure 3, infarcted rats had exacerbated LVEDP and decreased +dP/dt, −dP/dt, SW, and CO as a response to sudden afterload increases. Mechanisms associated with changes in cardiac performance are not fully clarified, but they may be linked to an altered handling Ca^2+^ and myofilament Ca^2+^ sensitivity (Pfeffer and Braunwald, 1990). Moreover, post-infarct ventricular dilatation is shown to be limiting for the intracavitary pressure development, as defined by the Laplace (Pfeffer and Braunwald, 1990; dos Santos et al., 2013). Importantly, LLLT had no effect on the functional cardiac abnormalities.

To our knowledge, infarct size has been the main variable affected by LLLT, and many studies have shown a minor injury size with several weeks post-LLLT application (Oron et al., 2001a,b; Yaakobi et al., 2001; Yang et al., 2011). It is hard to understand the differences of our findings to previously studies. A key reason may be the randomization, in which we have only included animals with large infarcts. The comparison of experimental groups that have a similar infarct size at baseline is a critical issue to avoid the causality of results and has not been controlled in previous studies. Thus, while it may be understood that the LLLT lead to a lower infarct size, it is possible also that rats with lower infarct sizes have been included in the LLLT-treated group. Moreover, previous investigations have only carried out a cross-sectional analysis (Oron et al., 2001a; Yaakobi et al., 2001; Yang et al., 2011), in which the causality cannot be determined. In this regard, we have analyzed the longitudinal repercussion of LLLT (Table 2) to clarify whether infarct size at baseline (3 days) changed over time as an effect of phototherapy. Other issues that show be investigated to understand the differences in our findings for previously studies are (i) the differences in irradiation parameters and (ii) approach to analyzing the infarct size (e.g., histomorphometric or echocardiographic).

Cardioprotective effects of LLLT are often attributed to angiogenic factors in a wide range of tissues (Dourado et al., 2011; Feng et al., 2012; Cury et al., 2013), including the ischemic myocardium (Tuby et al., 2006). Thus, there are findings showing greater pro-angiogenic stimuli (e.g., VEGF expression) in infarcted hearts that received LLLT only in the early MI (Mirsky et al., 2002; Zhang et al., 2010). In our study, there was no increased VEGF expression and its well-known downstream—Akt with 3 days and 5 weeks post-MI. It is shown to be reported that time of analysis of VEGF post-infarction may be a reason for our findings. Zhao et al. (2010) conducted experiments on infarcted rats to investigate the temporal expression of angiogenic factors. The authors observed a significant increase in the VEGF protein levels at the border zone only during day one post-MI and with subsequent decline in 28 days. Consequently, we cannot exclude an effect of LLLT on angiogenic VEGF signaling because our analysis may have been influenced by the timeline.

In summary, our findings illustrate that LLLT improves LV systolic function in the early post-infarction cardiac remodeling. However, this beneficial effect may be dependent on the maintenance of phototherapy. Long-term studies with LLLT application are required to establish whether these effects ultimately translate into improved cardiac remodeling.

## Author contributions

MM, drafted the work and substantially contributed to work design, as well as, acquired, analyzed and interpreted all data. EA, drafted the work and substantially contributed to work design, as well as, acquired, analyzed and interpreted all data. JS, drafted the work and substantially contributed to work design, as well as, acquired, analyzed and interpreted the all data s and protein expression protocols. Pd, laser protocol and dosage. RA, laser protocol and dosage. FP, drafted the work and substantially contributed to work design, as well as, acquired, analyzed and interpreted all data. RF, performed experiments and protein expression protocols. JM, Echocardiogram analysis. SV, drafted the work and substantially contributed to work design, as well as, acquired, analyzed and interpreted all data. VG, drafted the work and substantially contributed to work design, as well as, acquired, analyzed and interpreted all data. Md, performed experiments and protein expression protocols. AY, performed experiments and histological analysis. MC, performed experiments and histological analysis. Rd, drafted the work and substantially contributed to work design, as well as, acquired, analyzed and interpreted all data. DB, performed experiments and protein expression protocols. Bd, performed experiments and protein expression protocols. PT, oversaw the design and performance of the experiments, analyzed data, interpreted the results of the experiments and edited the final format of the manuscript. AS, oversaw the design and performance of the experiments, analyzed data, interpreted the results of the experiments, edited and revised manuscript. All authors revised the work critically, approved the final version to be published and declared accountable for all aspects of the work.

## Funding

This study was supported by the Conselho Nacional de Desenvolvimento Científico e Tecnológico (grant #400851/2014-8) and FAPESP (Grants: 09-54225/8; 15/11028-9).

### Conflict of interest statement

The authors declare that the research was conducted in the absence of any commercial or financial relationships that could be construed as a potential conflict of interest.

## Abbreviations

MI: Myocardial infarction
HF: Heart failure
LLLT: Low-level laser therapy
LV: Left ventricular
ATP: Adenosine triphosphate
NHI: National Institutes of Health
MI-LLLT: Infarcted rats submitted with laser
MI-N: Infarcted rats non-treated with laser
AlGaInP: Aluminum Indium Gallium Phosphorus
EDP: End diastolic pressure
+d*P*/d*t*: Positive derivatives of the developed pressure
-d*P*/d*t*: Negative derivatives of the developed pressure
SV: Stroke volume
SP: Systolic pressure
SWI: Stroke work index
CO: Cardiac output
VEGF: Vascular endothelial grow factor.

## References

[B1] Ad N. Oron U. (2001). Impact of low level laser irradiation on infarct size in the rat following myocardial infarction. Int. J. Cardiol. 80, 109–116. 10.1016/S0167-5273(01)00503-4 11578700

[B2] Albertini R. Aimbire F. Silva J. Jr. Costa M. S. (2008). Cytokine mRNA expression is decreased in the subplantar muscle of rat paw subjected to carrageenan-induced inflammation after low-level laser therapy. Photomed. Laser Surg. 26, 19–24. 10.1089/pho.2007.2119 18248157

[B3] Antonio E. L. Serra A. J. dos Santos A. A. Vieira S. S. Silva J. M. Yoshizaki A.. (2015). Are there gender differences in left ventricular remodeling after myocardial infarction in rats? Rev. Bras. Cir. Cardiovasc. 30, 70–76. 10.5935/1678-741.20140093 25859870 PMC4389530

[B4] AVMA Panel on Euthanasia. American Veterinary Medical Association (2001). 2000 Report of the AVMA Panel on Euthanasia. J. Am. Vet. Med. Assoc. 218, 669–696. 10.2460/javma.2001.218.669 11280396

[B5] Carlos F. P. Gradinetti V. Manchini M. de Tarso C. C. P. Silva J. A. Jr. Girardi A. C.. (2016). Role of low-level laser therapy on the cardiac remodeling after myocardial infarction: a systematic review of experimental studies. Life Sci. 151, 109–114. 10.1016/j.lfs.2016.02.058 26930372

[B6] Cury V. Moretti A. I. Assis L. Bossini P. Crusca J. S. Neto C. B. (2013). Low level laser therapy increases angiogenesis in a model of ischemic skin flap in rats mediated by VEGF, HIF-1a and MMP-2. J. Photochem. Photobiol. B 125, 164–170. 10.1016/j.jphotobiol.2013.06.004 23831843 PMC3759230

[B7] dos Santos L. Antonio E. L. Souza A. F. M. Tucci P. F. J. (2010). Use of afterload hemodynamic stress as a practical method for assessing cardiac performance in rats with heart failure. Can. J. Physiol. Pharmacol. 88, 724–732. 10.1139/y10-062 20651820

[B8] dos Santos L. Gonçalves G. A. Davel A. P. Santos A. A. Krieger J. E. Rossoni L. V.. (2013). Cell therapy prevents structural, functional and molecular remodeling of remote non-infarcted myocardium. Int. J. Cardiol. 168, 3829–3836. 10.1016/j.ijcard.2013.06.026 23849970

[B9] Dourado D. M. Fa'vero S. Matias R. Carvalho P. T. da Cruz-Hofling M. A. (2011). Low-level laser therapy promotes vascular endothelial growth factor receptor-1 expression in endothelial and nonendothelial cells of mice gastrocnemius exposed to snake venom. Photochem. Photobiol. 87, 418–426. 10.1111/j.1751-1097.2010.00878.x 21166811

[B10] Feng J. Zhang Y. Xing D. (2012). Low-power laser irradiation (LPLI) promotes VEGF expression and vascular endothelial cell proliferation through the activation of ERK/Sp1 pathway. Cell Signal. 24, 1116–1125. 10.1016/j.cellsig.2012.01.013 22326662

[B11] Fletcher P. Pfeffer J. Pfeffer M. Braunwald E. (1981). Left ventricular diastolic pressure-volume relations in rats with healed myocardial infarction. Effects on systolic function. Circ. Res. 49, 618–626. 10.1161/01.RES.49.3.618 7261261

[B12] Francis J. Weiss R. M. Wei S. G. Johnson A. K. Felder R. B. (2001). Progression of heart failure after myocardial infarction in the rat. Am. J. Physiol. Regul. Integr. Comp. Physiol. 281, R1734–R1745. 11641147 10.1152/ajpregu.2001.281.5.R1734

[B13] Grosman-Rimon L. Billia F. Fuks A. Jacobs I. A. McDonald M. Rao V.. (2016). New therapy, new challenges: the effects of long-term continuous flow left ventricular assist device on inflammation. Int. J. Cardiol. 215, 424–430. 10.1016/j.ijcard.2016.04.133 27131263

[B14] Huang Y. Y. Sharma S. K. Carroll J. Hamblin M. R. (2011). Biphasic dose response in low level light therapy -an update. Dose Response 9, 602–618. 10.2203/dose-response.09-027.Hamblin 22461763 PMC3315174

[B15] Kolseth S. M. Rolim N. P. Salvesen Ø. Nordhaug D. O. Wahba A. Høydal M. A. (2014). Levosimendan improves contractility *in vivo* and *in vitro* in a rodent model of post-myocardial infarction heart failure. Acta. Physiol. 210, 865–874. 10.1111/apha.12248 24495280

[B16] Manchini M. T. Serra A. J. Feliciano Rdos S. Santana E. T. Antônio E. L. de Tarso Camillo de Carvalho P.. (2014). Amelioration of cardiac function and activation of anti-inflammatory vasoactive peptides expression in the rat myocardium by low level laser therapy. PLoS ONE 9:e101270. 10.1371/journal.pone.0101270 24991808 PMC4081549

[B17] Mirsky N. Krispel Y. Shoshany Y. Maltz L. Oron U. (2002). Promotion of angiogenesis by low energy laser irradiation. Antioxid. Redox Signal. 4, 785–790. 10.1089/152308602760598936 12470506

[B18] Oron U. Yaakobi T. Oron A. Hayam G. Gepstein L. Rubin O.. (2001a). Attenuation of infarct size in rats and dogs after myocardial infarction by low-energy laser irradiation. Lasers Surg. Med. 28, 204–211. 10.1002/lsm.1039 11295753

[B19] Oron U. Yaakobi T. Oron A. Mordechovitz D. Shofti R. Hayam G.. (2001b). Low-energy laser irradiation reduces formation of scar tissue after myocardial infarction in rats and dogs. Circulation 103, 296–301. 10.1161/01.CIR.103.2.296 11208692

[B20] Peplow P. V. Chung T. Y. Baxter G. D. (2012). Photodynamic modulation of wound healing: a review of human and animal studies. Photomed. Laser Surg. 30, 118–114. 10.1089/pho.2011.3142 22283621

[B21] Pfeffer M. A. Braunwald E. (1990). Ventricular remodeling after myocardial infarction. Experimental observations and clinical implications. Circulation 81, 1161–1172. 2138525 10.1161/01.cir.81.4.1161

[B22] Pires D. Xavier M. Araujo T. R. Junior J. A. S. Aimbire F. Albertini R. (2011). Low-level laser therapy (LLLT; 780 nm) acts differently on mRNA expression of anti- and pro-inflammatory mediators in an experimental model of collagenase-induced tendinitis in rat. Lasers Med. Sci. 26, 85–94. 10.1007/s10103-010-0811-z 20737183

[B23] Serra A. J. Santos M. H. Bocalini D. S. Antônio E. L. Levy R. F. Santos A. A.. (2010). Exercise training inhibits inflammatory cytokines and more than prevents myocardial dysfunction in rats with sustained beta-adrenergic hyperactivity. J. Physiol. 588, 2431–2442. 10.1113/jphysiol.2010.187310 20442263 PMC2915518

[B24] Serra A. J. Tucci P. J. (2016). How should experimental myocardial infarction size be reported? Int. J. Cardiol. 214, 189–190. 10.1016/j.ijcard.2016.03.151 27064639

[B25] Silva J. A. Jr. Santana E. T. Manchini M. T. Antônio E. L. Bocalini D. S. Krieger J. E.. (2014). Exercise training can prevent cardiac hypertrophy induced by sympathetic hyperactivity with modulation of kallikrein-kinin pathway and angiogenesis. PLoS ONE 9:e91017. 10.1371/journal.pone.0091017 24614810 PMC3948752

[B26] Sofia R. R. Serra A. J. Silva J. A. Jr. Antonio E. L. Manchini M. T. Oliveira F. A.. (2014). Gender-based differences in cardiac remodeling and ILK expression after myocardial infarction. Arq. Bras. Cardiol. 103, 124–130. 10.5935/abc.20140113 25098374 PMC4150663

[B27] Tuby H. Maltz L. Oron U. (2006). Modulations of VEGF and iNOS in the rat heart by low level laser therapy are associated with cardioprotection and enhanced angiogenesis. Lasers Surg. Med. 38, 682–688. 10.1002/lsm.20377 16800001

[B28] Tuby H. Maltz L. Oron U. (2008). Implantation of low-level laser irradiated mesenchymal stem cells into the infarcted rat heart is associated with reduction in infarct size and enhanced angiogenesis. Photomed. Laser Surg. 27, 227–233. 10.1089/pho.2008.2272 19382832

[B29] Whelan R. S. Kaplinskiy V. Kitsis R. N. (2010). Cell death in the pathogenesis of heart disease: mechanisms and significance. Annu. Rev. Physiol. 72, 19–44. 10.1146/annurev.physiol.010908.163111 20148665 PMC12973270

[B30] Whittaker P. Patterson M. J. (2000). Ventricular remodeling after acute myocardial infarction: effect of low-intensity laser irradiation. Lasers Surg. Med. 27, 29–38. 10.1002/1096-9101(2000)27:1<29::AID-LSM4>3.0.CO;2-8 10918290

[B31] Yaakobi T. Shoshany Y. Levkovitz S. Rubin O. Ben Haim S. A. Oron U. (2001). Long-term effect of low energy laser irradiation on infarction and reperfusion injury in the rat heart. J. Appl. Physiol. 90, 2411–2419. 11356808 10.1152/jappl.2001.90.6.2411

[B32] Yancy C. W. Jessup M. Bozkurt B. Butler J. Casey D. E. Jr. Drazner M. H.. (2013). 2013 ACCF/AHA guideline for the management of heart failure: a report of the American College of Cardiology Foundation/American Heart Association Task Force on Practice Guidelines. J. Am. Coll. Cardiol. 62, e147–e239. 10.1016/j.jacc.2013.05.019 23747642

[B33] Yang Z. Wu Y. Zhang H. Jin P. Wang W. Hou J.. (2011). Low-level laser irradiation alters cardiac cytokine expression following acute myocardial infarction: a potential mechanism for laser therapy. Photomed. Laser Surg. 29, 391–398. 10.1089/pho.2010.2866 21348574

[B34] Zhang H. Hou J-F., Shen, Y. Wang W. Wei Y-J., Hu, S. (2010). Low level laser irradiation precondition to create friendly milieu of infarcted myocardium and enhance early survival of transplanted bone marrow cells. J. Cell. Mol. Med. 14, 1975–1987. 10.1111/j.1582-4934.2009.00886.x 19725921 PMC3823279

[B35] Zhao T. Zhao W. Chen Y. Ahokas R. A. Sun Y. (2010). Vascular endothelial growth fator (VEGF)-A: role on cardiac angiogenesis following myocardial infarction. Microvasc Res. 80, 188–194. 10.1016/j.mvr.2010.03.014 20362592 PMC3628688

[B36] Ziff O. J. Covic A. Goldsmith D. (2016). Calibrating the impact of dual RAAS blockade on the heart and the kidney - balancing risks and benefits. Int. J. Clin. Pract. 70, 537–553. 10.1111/ijcp.12803 27278080

